# Clozapine-Induced Mitochondria Alterations and Inflammation in Brain and Insulin-Responsive Cells

**DOI:** 10.1371/journal.pone.0059012

**Published:** 2013-03-20

**Authors:** Verόnica Contreras-Shannon, Dylan L. Heart, R. Madelaine Paredes, Erica Navaira, Gabriel Catano, Shivani Kaushal Maffi, Consuelo Walss-Bass

**Affiliations:** 1 Department of Biological Sciences, Saint Mary's University, San Antonio, Texas, United States of America; 2 Department of Psychiatry, University of Texas Health Science Center, San Antonio, Texas, United States of America; 3 Department of Medicine, and the Veterans Administration Center for Personalized Medicine, South Texas Veterans Health Care System, University of Texas Health Science Center, San Antonio, Texas, United States of America; 4 Department of Molecular Medicine, University of Texas Health Science Center, San Antonio, Texas, United States of America; 5 Medical Research Division, Regional Academic Health Center-Edinburg, Edinburg, Texas, United States of America; Université Joseph Fourier, France

## Abstract

**Background:**

Metabolic syndrome (MetS) is a constellation of factors including abdominal obesity, hyperglycemia, dyslipidemias, and hypertension that increase morbidity and mortality from diabetes and cardiovascular diseases and affects more than a third of the population in the US. Clozapine, an atypical antipsychotic used for the treatment of schizophrenia, has been found to cause drug-induced metabolic syndrome (DIMS) and may be a useful tool for studying cellular and molecular changes associated with MetS and DIMS. Mitochondria dysfunction, oxidative stress and inflammation are mechanisms proposed for the development of clozapine-related DIMS. In this study, the effects of clozapine on mitochondrial function and inflammation in insulin responsive and obesity-associated cultured cell lines were examined.

**Methodology/Principal Findings:**

Cultured mouse myoblasts (C2C12), adipocytes (3T3-L1), hepatocytes (FL-83B), and monocytes (RAW 264.7) were treated with 0, 25, 50 and 75 µM clozapine for 24 hours. The mitochondrial selective probe TMRM was used to assess membrane potential and morphology. ATP levels from cell lysates were determined by bioluminescence assay. Cytokine levels in cell supernatants were assessed using a multiplex array. Clozapine was found to alter mitochondria morphology, membrane potential, and volume, and reduce ATP levels in all cell lines. Clozapine also significantly induced the production of proinflammatory cytokines IL-6, GM-CSF and IL12-p70, and this response was particularly robust in the monocyte cell line.

**Conclusions/Significance:**

Clozapine damages mitochondria and promotes inflammation in insulin responsive cells and obesity-associated cell types. These phenomena are closely associated with changes observed in human and animal studies of MetS, obesity, insulin resistance, and diabetes. Therefore, the use of clozapine in DIMS may be an important and relevant tool for investigating cellular and molecular changes associated with the development of these diseases in the general population.

## Introduction

This study addresses the cellular and molecular basis of a highly significant public health problem: metabolic syndrome (MetS). MetS is a constellation of factors including abdominal obesity, hyperglycemia, dyslipidemias, and hypertension that increase morbidity and mortality from diabetes and cardiovascular diseases [1], [2], [3], [4]. According to the most recent National Health Statistics Reports, approximately 34% of the adult population in the U.S. meets the criteria for having MetS [5]. Recent estimates indicate that independent of cardiovascular disease, risk factors associated with MetS cost an estimated $80 billion annually [6] and are projected to increase between 59% and 157% by 2020 [7]. Because of this significant health problem and its economic burden, there is a great need to better understand the cellular and molecular basis of MetS.

There is an abundance of studies investigating MetS, obesity, and diabetes in human and animal model systems. These models are complex, heterogenous systems representing multiple cellular, biochemical, molecular, and physiological pathways. In this study, we utilize clozapine as a tool for studying drug-induced metabolic syndrome (DIMS) in cultured mammalian cell types that are typically associated with MetS. Cultured cell models provide a straightforward system for detecting key cellular and molecular changes that may be associated with MetS. Clozapine is an atypical antipsychotic that is highly efficacious for the treatment of schizophrenia. However, along with most atypical antipsychotics, clozapine has been found to cause DIMS, giving rise to adverse metabolic side effects such as obesity and increased diabetes risk [8], [9]. The underlying biological causes of clozapine-associated DIMS are unknown. There is a growing consensus in the obesity and diabetes fields that understanding the mechanisms responsible for the adverse metabolic effects of atypical antipsychotics may shed an important light on the origin of MetS, and this is the rationale for using this model in the current study.

There are three interrelated hypotheses that have been proposed to explain antipsychotic-induced metabolic side effects. First, these drugs negatively affect the proper functioning of mitochondria [10], [11], [12], [13], [14]. Specifically, these drugs may alter the function of key metabolic enzymes and thus negatively affect carbon metabolism and/or electron transport during oxidative phosphorylation. Clozapine has been shown to promote the oxidation of mitochondrial proteins involved in energy metabolism in neuroblastoma cells and in lymphoblastoid cells of schizophrenia patients [10], [11]. Oxidized proteins included enzymes important in carbon metabolism such as pyruvate kinase and mitochondrial malate dehydrogenase. Analyses of rat or mice brains have shown that clozapine alters mitochondrial function, energy metabolism, and expression of mitochondrial proteins belonging to the electron transport chain and oxidative phosphorylation pathway, such as succinate dehydrogenase and cytochrome oxidase [12], [13]. In addition, alterations in electron transport were demonstrated in peripheral blood cells of patients taking atypical antipsychotics [14]. Second, these drugs may cause increased oxidative stress in cells and tissues [15], [16], [17]. In addition to direct protein oxidation, antipsychotic treatment has been associated with increased production of reactive oxygen species (ROS) and antioxidant proteins. In a study of patients undergoing long-term clozapine treatment, there were elevated levels of the antioxidant enzyme superoxide dismutase in red blood cells [18]. Further evidence of clozapine-induced production of reactive oxygen species (ROS) was demonstrated in rat whole blood [19] and rat brain [16], [17]. Third, these drugs promote inflammation [20], [21], [22]. There is evidence to suggest that clozapine influences the production of several cytokines and/or cytokine receptors that modulate immunological responses [20], [21], [22]. In stimulated blood from healthy donors, clozapine treatment increased levels of IL-4 and IL-17 [13]. In a study which administered clozapine to schizophrenia patients over a six week period, plasma levels of cytokines, including TNF-α, sTNFR-1, sTNFR-2, IL-6, and sIL-2R, were found to increase significantly over the treatment time [14]. Lastly, it is important to note the interplay between these three proposed mechanisms. Mitochondria that are damaged are known to produce increased levels of ROS and initiate inflammation; oxidative stress itself can damage mitochondria and promote an inflammatory response [23], [24]. Similarly, a pro-inflammatory state contributes to increased ROS production and can negatively affect mitochondria function, either directly, or through oxidative stress [24], [25].

These same mechanisms of mitochondria dysfunction, oxidative stress and inflammation are also attributed to the development of obesity, insulin resistance and other symptoms of diabetes and MetS [26], [27], [28]. Patients who suffer from obesity, insulin resistance or diabetes have been found to have dysfunctional mitochondria [29]. In these patients, electron transport [30] and oxidative phosphorylation [31], [32] are altered. Patients with metabolic disease also have alterations in mitochondria morphology or number [33]. Further, altered mitochondria dynamics, as well as oxidative stress have been associated with altered muscle metabolism and insulin resistance in mouse skeletal muscle [22]. Regarding oxidative stress, there are several studies which describe in detail the role of ROS and decreased antioxidant capacity in dyslipidemia, obesity, insulin resistance, diabetes and MetS [34], [35]. In many disease models, including diabetes, ROS promote a pro-inflammatory environment. It has been shown that mitochondrial ROS trigger expression of pro-inflammatory cytokines as a result of the oxidative stress process [36]. Therefore, it is not surprising that inflammation has also been shown to play a key role in obesity and diabetes [27]. Previous studies have shown that chronic activation of intracellular pro-inflammatory pathways within insulin target cells can lead to obesity-related insulin resistance [28]. Further, inflammation and cytokine production are an extracellular source of ROS. Thus, ROS and inflammation are inextricably and cyclically linked to metabolic disease and metabolic dysfunction. Not surprisingly, elevated cytokines have been found in the serum of patients suffering from diseases where inflammation is a key factor [37]. Inflammatory cytokines such as IL-6, IL-1β, MCP-1 and TNF- α, produced by fat, liver, muscle, and inflammatory cells, have been described as key players in obesity, insulin resistance and diabetes [28]. Patients with MetS have increased circulating levels of numerous cytokines such as IL-2, IL-4, IL-5, IL-12, IL-12, and INF-γ, while patients with type II diabetes have circulating T cells which produce increased levels of IL-17 and IFN-γ [38]. Thus, in this study, we explore whether clozapine-associated DIMS may be attributed to mitochondria dysfunction, oxidative stress and inflammation.

Importantly, much of the work investigating the mechanisms responsible for antipsychotic-induced metabolic side effects has primarily focused on the brain and blood, for the purposes of understanding how these drugs affect their target population, schizophrenia patients. While those investigations are meaningful and important, there are two problems with this approach. First, they neglect the biology of obesity and diabetes. The major contributing factor in type II diabetes and obesity is insulin resistance [39]. Insulin resistance is thought to be mediated by the release of pro-inflammatory molecules and other mediators from adipocytes and inflammatory cells which alter insulin response in fat and muscle tissue [28]. Secondly, this approach is short sighted; DIMS can be an important tool for studying cellular and molecular changes that lead to MetS in the greater population, not just within the psychiatric milieu. For these reasons, in addition to human neuroblastoma cells, the present study examines the effects of clozapine on insulin responsive and obesity-associated cell types: cultured mouse fat, muscle, liver and inflammatory cell lines. The mouse 3T3-L1 (herein referred to as 3T3) adipocyte, C2C12 myoblast, FL83B hepatocyte, and RAW264.7 (herein referred to as RAW) monocyte cell lines, representing fat, muscle, liver, and inflammatory cells, respectively, were used in order to directly assess the cellular and molecular effects of clozapine treatment within a homogenous cell culture system. In this study, we specifically examine the effects of clozapine on mitochondria morphology and function as well as on the production of proinflammatory cytokines. Understanding how clozapine increases risk for MetS may provide insight into the development of metabolic disease in the general population.

## Results

### Clozapine-induced mitochondria changes in neuroblastoma cells

**Figure 1:**
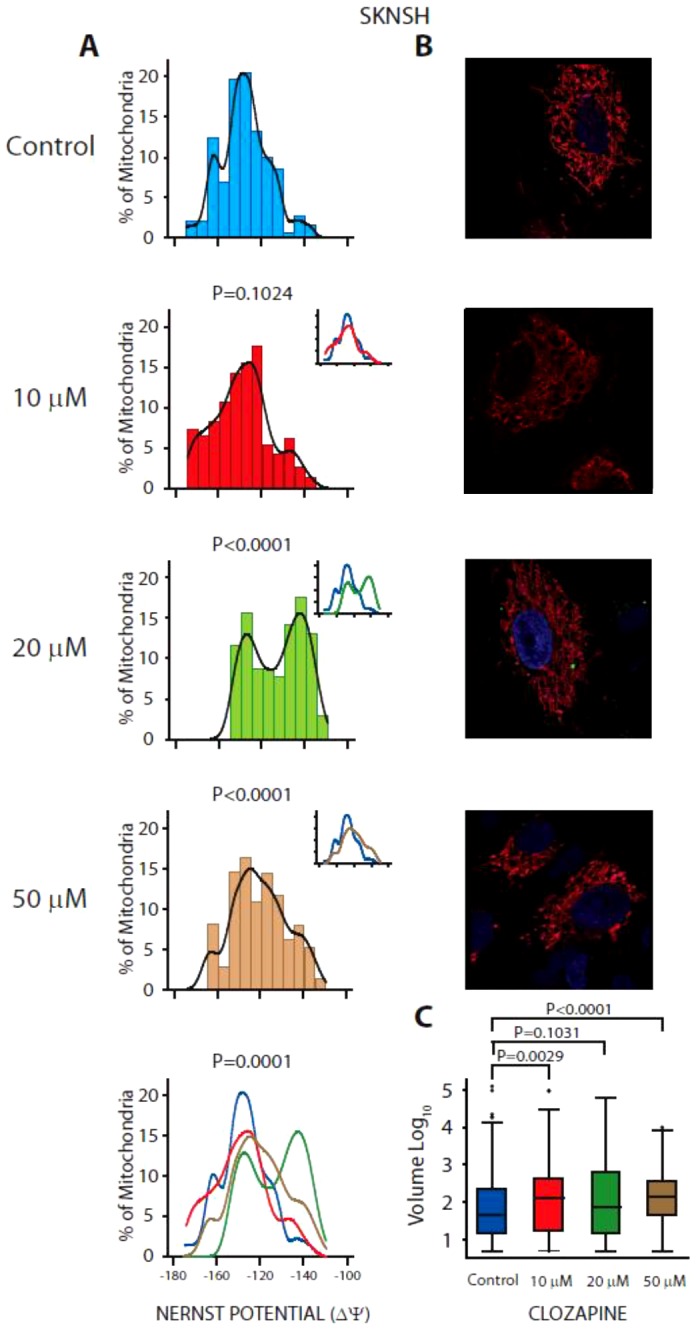
The effect of clozapine treatment on mitochondria. A) membrane potential, B) morphology, and C) volume in SKNSH neuroblastoma cells. SKNSH cells were treated with 0, 10, 20 or 50 µM clozapine for 24 hours and then incubated with TMRM for 20 minutes at 37°C with 5% CO_2_. Confocal micrographs analyzed for mitochondrial membrane potential and volume using the Nernst Potential MulPro2D plug-in for Image J software.

Previous studies demonstrated that clozapine treatment in neuroblastoma cells did not alter cell viability or induce apoptosis, but did increase ROS and oxidized mitochondrial proteins important for energy metabolism [11]. To further explore the effects of clozapine on mitochondria functions, the effect of clozapine on mitochondria morphology, membrane potential, and volume in neuroblastoma cells was assessed here. After treatment with 10, 20 and 50 µM clozapine or vehicle only, neuroblastoma cells were incubated with the mitochondria-selective probe, tetramethylrhodamine, methyl ester, perchlorate (TMRM) and imaged by confocal microscopy. At the highest concentration of clozapine (50 µM), the mitochondria were found to be mostly punctate and round when compared to a mixed population of reticular filamentous arrangement in the perinuclear area along with a smaller puncta cytoplasmic mitochondrial distribution observed in the control cells, and in cells treated with 10 or 20 µM clozapine (Figure 1B). The analyses of the distribution of TMRM fluorescence provided a measure of the mitochondria membrane potential, which under normal conditions varies between −180 to −120 mV, though more than 24 percent of healthy mitochondria exhibit a membrane potential between (−180 and −140 ΔΨ). Treatment of neuroblastoma cells with clozapine shifted cells from their normal membrane potential distribution (Figure 1A and Table S1). Above 10 µM clozapine, the distribution of mitochondrial membrane potential is observed in two or more distinct populations. A group of mitochondria still possess high membrane potential, however, between 48 to 65% of mitochondria exhibit significantly decreased membrane potential (p≤0.0001), compared to the control untreated cells. Analyses of the percent change in Nernst potential above indicated thresholds, and the percent distribution above the median of the control, to represent the shift in the medians after treatment, are shown in Table S1. In addition, measurements of central tendency (median and mean) and dispersion (IQR and SD) for the distribution of the membrane potential and mitochondria volume data across the various concentrations of clozapine used can be found in Table S2. Lastly, relative to the control cells, the mean volume of mitochondria increased approximately 1.5-fold at 10 µM (p = 0.0029) and over two-fold at 50 µM clozapine (p<0.0001) (Figure 1C). In the box plot, outliers seen above control and 10 µM clozapine represent the large framework of contiguous mitochondrial networks, between Log_10_(4–5); the majority of the smaller mitochondrial fragments fall within the inter-quartile range, i.e. between the 25^th^–75^th^ percentiles of the total values. The horizontal line across the box shows the median value of mitochondrial volume in each group, flanked by whiskers which are 1.5 times the interquartile range.

### Clozapine-induced mitochondria changes in insulin-responsive cells

**Figure 2:**
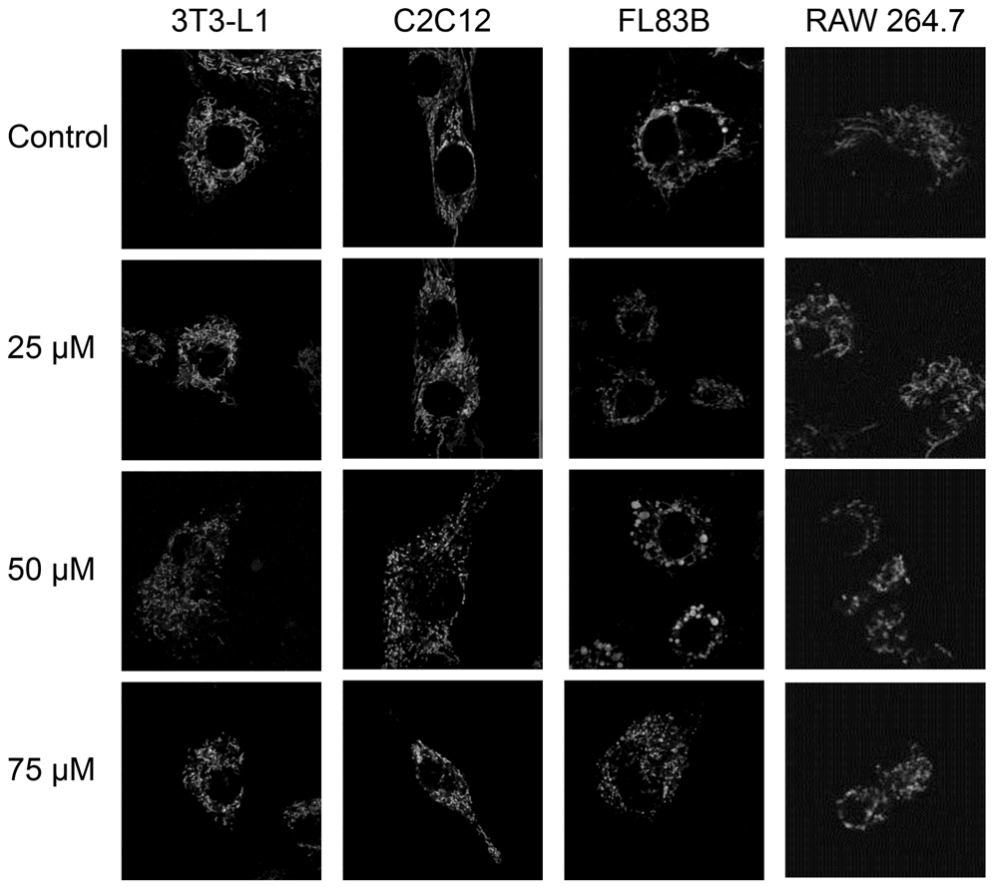
The effect of clozapine treatment on mitochondria morphology in 3T3-L1, C2C12, FL83B and RAW 264.7 cells. Cells were treated with 0, 25, 50 or 75 µM clozapine for 24 hours and then incubated with TMRM for 20 minutes at 37°C with 5% CO_2_. Mitochondria morphology was observed by fluorescent confocal microscopy. All images were taken using a 63x/1.4 Oil objective; images of RAW 264.7 cells are digitally enlarged 3× since these cells are smaller and appear to shrink with clozapine treatment.

After observing the effects of clozapine on the mitochondria of neuroblastoma cells, we were interested in examining whether similar changes occurred in cell types associated with whole-body metabolism: fat, muscle, liver and inflammatory cells. Changes in each of these cells types have been closely associated with metabolic alterations such as insulin resistance, obesity and diabetes [28]. The mouse cell lines which were used were: fat represented by 3T3 adipocytes, muscle represented by C2C12 myoblasts, liver represented by FL83B hepatocytes, and inflammatory cells represented by RAW264.7 monocytes. To determine whether clozapine affected these cells in the same fashion as neuroblastoma cells, we first assessed whether clozapine treatment affected cell viability. After treatment with 25, 50 and 75 µM clozapine or vehicle only, the effect of clozapine, at each dose, for each cell line, on viability was determined by neutral red uptake assay. Similar to the neuroblastoma cells, the concentrations of clozapine used did not cause significant differences in cell viability in three of the four cell lines. At the highest concentration of clozapine (75 µM), RAW monocyte viability decreased significantly relative to the control cells (p = 0.03). The percentage of viable monocytes, relative to the untreated control, was 62.1±11.8%. We then examined whether clozapine also induced changes in mitochondria morphology, membrane potential, and mitochondria volume in the insulin-responsive cell types as these types of changes are thought to underlie the pathogenesis of several metabolic diseases [29]. Treatment with 25, 50 or 75 µM clozapine, or vehicle only, for 24 hours, was followed by incubation with TMRM and confocal microscopic imaging. Similar to the neuroblastoma cells, each of the cell lines show a transition of mitochondrial morphology and distribution from filamentous network to a more fissioned mitochondria, having a punctate appearance at the highest clozapine concentration (Figure 2).

**Figure 3:**
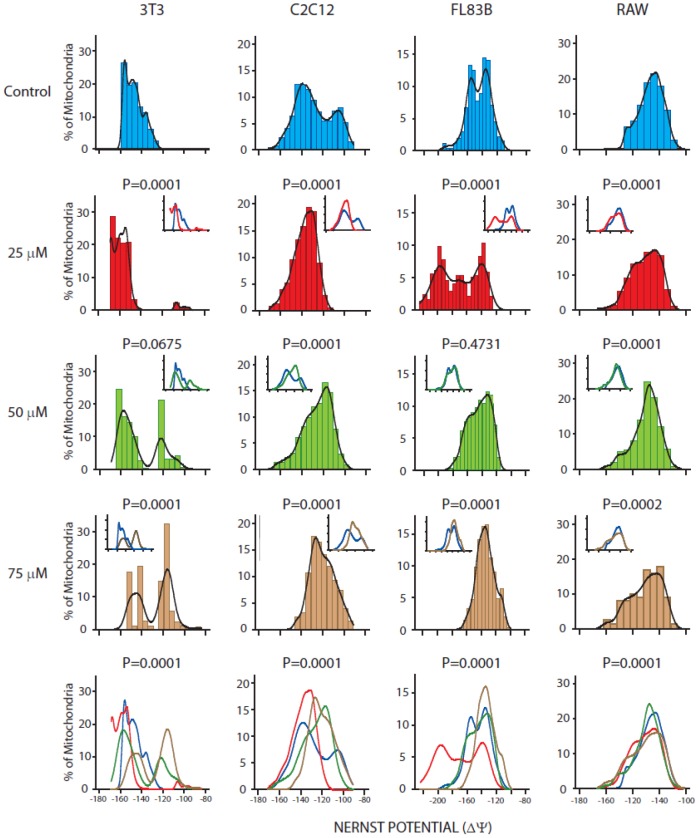
The effect of clozapine treatment on mitochondria membrane potential in 3T3-L1, C2C12, FL83B and RAW 264.7 cells. Cells were treated with 0, 25, 50 and 75 µM clozapine for 24 hours followed by incubation with TMRM for 20 minutes at 37°C with 5% CO_2_. The fluorescence intensity of TMRM was monitored using confocal microscopy. Micrographs were analyzed for mitochondrial membrane potential using the Nernst Potential MulPro2D plug-in for Image J software.

In addition to the mitochondria morphological changes, treatment of the insulin-responsive cell lines with clozapine shifted cells from their normal mitochondria membrane potential distribution (Figure 3) to a more depolarized state, particularly at the higher concentrations used. Interestingly, each cell type showed a varied distribution pattern of mitochondrial membrane potential. As in the case for neuroblastoma cells, further analyses of the percent change in Nernst potential and the percent distribution above the median of the control due to treatment are shown in Table S1, as well as central tendency and dispersion data in Table S2. At 25 µM clozapine, about 43% of mitochondria in 3T3 preadipocytes get highly polarized by increasing their membrane potential compared to the untreated controls (p = 0.0001). However, mitochondrial membrane potential decreased significantly and clustered in two distinct populations (around −160 to −140 mV and −120 to −100 mV, respectively) at 75 µM clozapine compared to control cells (p = 0.0001). The C2C12 myoblasts showed an initial increase in mitochondrial membrane potential (about 18% of the Nernst values shifted to the left) at 25 µM clozapine relative to controls (p = 0.0001), followed by a shift in distribution curves of Nernst potential to the right, suggesting depolarization of a large % (between 18 to 34%) of mitochondria from −140 mV to potentials ranging between −130 to −110 mV, at both 50 and 75 µM clozapine (p = 0.0001). For the FL83B hepatocytes, the proportion of mitochondria with reduced membrane potential increased by 26% at 75 µM clozapine (p = 0.0001). Similarly, for the RAW monocytes at 75 µM clozapine, there was an increase of 8% in the population of depolarized mitochondria when compared to the control cells (p = 0.0001) (Figure 3).

**Figure 4:**
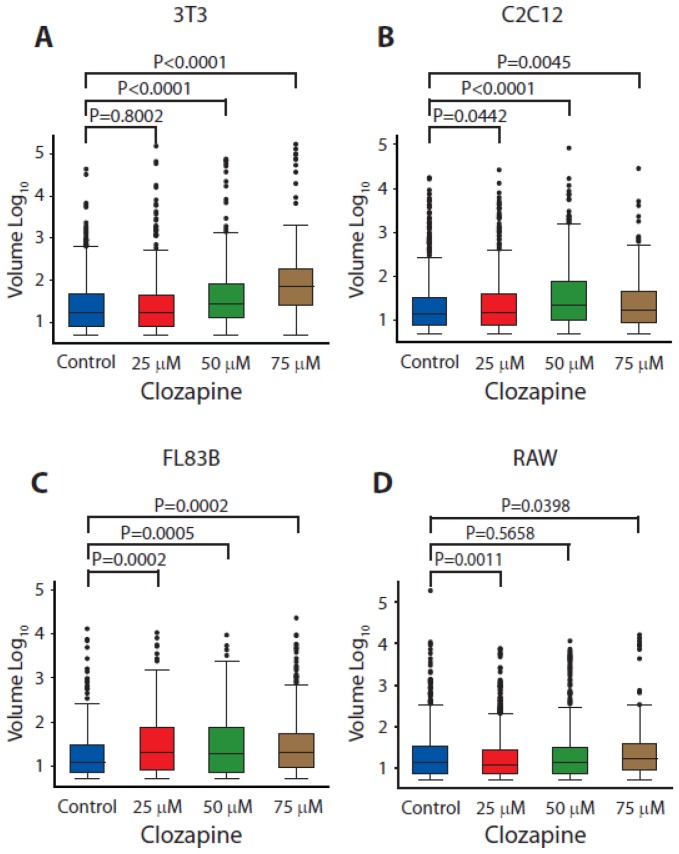
The effect of clozapine treatment on mitochondria volume in 3T3-L1, C2C12, FL83B and RAW 264.7 cells. Cells were treated with 0, 25, 50 and 75 µM clozapine for 24 hours followed by incubation with TMRM for 20 minutes at 37°C with 5% CO_2_. The fluorescence intensity of TMRM was assessed by confocal microscopy. Micrographs were analyzed for mitochondrial volume using the Nernst Potential MulPro2D plug-in for Image J software.

Mitochondria volumes increased with clozapine treatment for all four cell lines (Figure 4 and table S2). The 3T3 cells had mean mitochondria volumes that increased 70% and 230%, respectively, relative to the controls at 50 and 75 µM clozapine (p<0.0001; Figure 4A). The mean mitochondria volumes of C2C12 cells increased 12% (p = 0.04), 55% (p<0.0001), and 22% (p = 0.0045) relative to the control cells at 25, 50 and 75 µM clozapine, respectively (Figure 4B). FL83B cells had mitochondria volumes that increased 55% (p = 0.0002), 51% (p = 0.0005), and 38% (p = 0.0002) relative to the controls at 25, 50 and 75 µM clozapine, respectively (Figure 4C). At 25 µM clozapine, the mitochondria volume of RAW cells decreased 15% (p = 0.0011) but increased 26% (p = 0.04) at 75 µM clozapine, relative to controls (Figure 4D).

### The effect of clozapine treatment on ATP production

**Figure 5:**
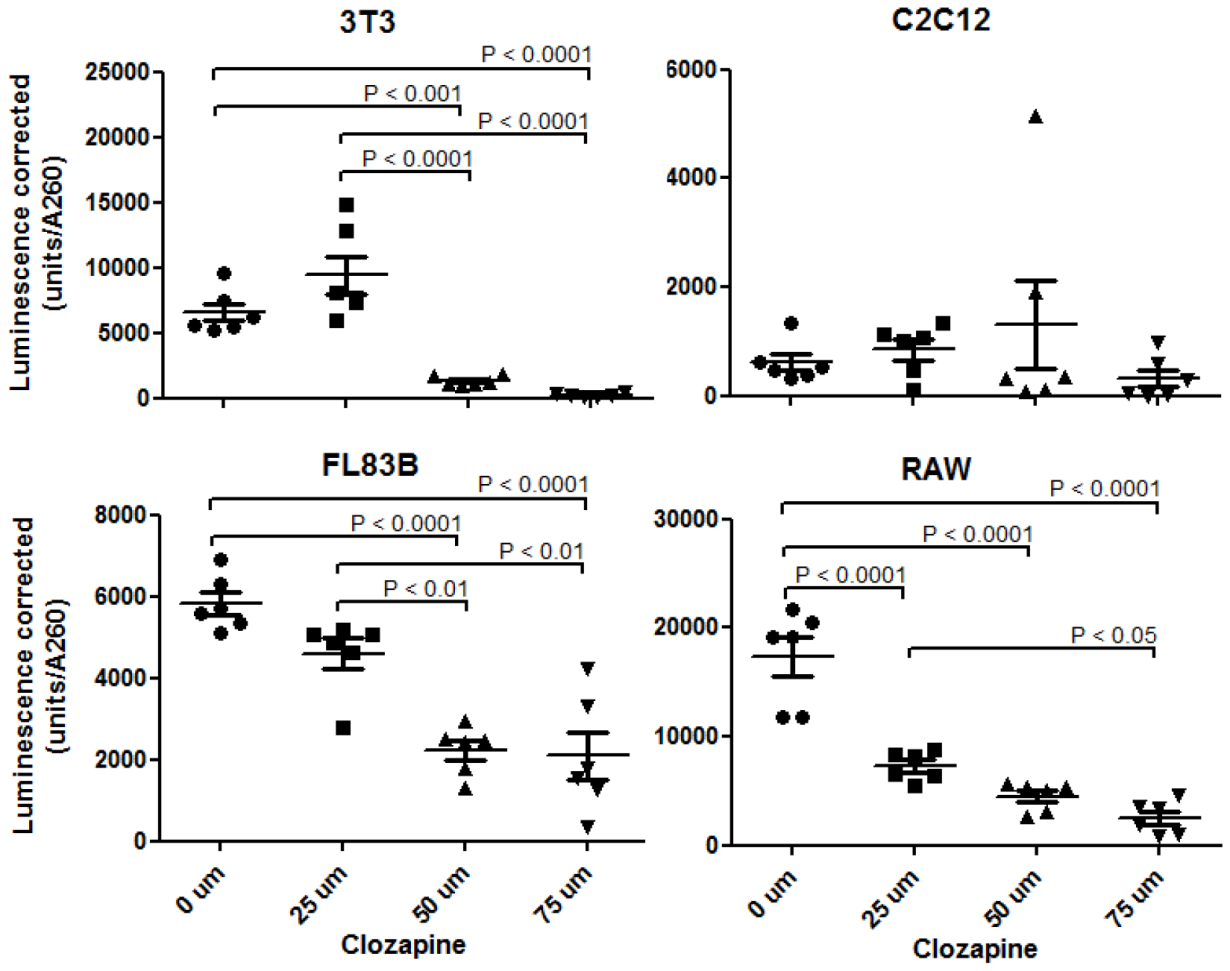
The effect of clozapine treatment on ATP production in 3T3-L1, C2C12, FL83B, and RAW264.7 cells. ATP levels were assessed using a bioluminescence assay in cells treated with 0, 25, 50 and 75 µM clozapine for 24 hours. Luminescence intensity corresponds to relative levels of ATP. Luminescence was corrected by using lysate A260 values to correct for the number of viable cells contributing to ATP levels. P values shown are based on a Bonferroni post-hoc test.

Changes in mitochondria morphology and membrane potential are associated with mitochondria dysfunction. To determine whether these changes had functional consequences, the effect of clozapine treatment on ATP levels was determined by bioluminescence assay. ATP levels significantly decreased with increasing doses of clozapine for the 3T3, FL83B and RAW cell lines (for all three, p<0.0001; Figure 5). At 50 and 75 µM clozapine, ATP levels in 3T3 cells were significantly reduced 80 and 96% (p<0.0001), respectively, relative to the controls, as well as 86 and 97% (p<0.0001) relative to the 25 µM dose of clozapine. ATP levels were significantly reduced 62% and 64% (p<0.0001), relative to the controls, at 50 and 75 µM clozapine, respectively, and 51 and 55% (p<0.01) relative to the 25 µM clozapine dose, respectively, in FL83B cells. Levels of ATP in RAW cells were significantly reduced 58, 74 and 85% (p<0.0001) relative to the controls at 25, 50 and 75 µM clozapine, respectively, and reduced 84% (p<0.05) relative to the 25 µM dose at 75 µM clozapine. Changes in ATP levels in C2C12 cells as a result of increasing clozapine concentration were not statistically significant.

### Clozapine-induced production of pro-inflammatory cytokines

**Figure 6:**
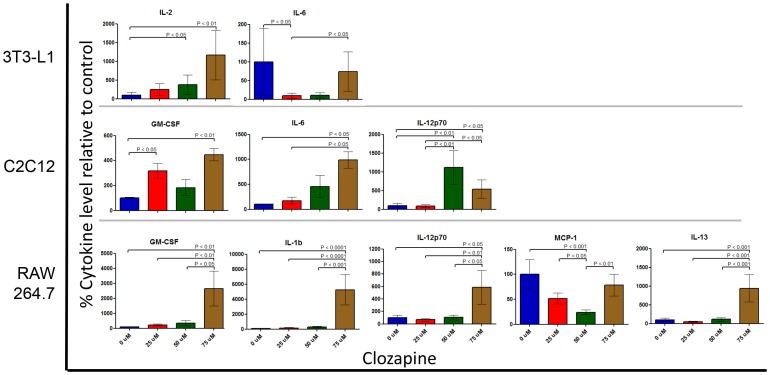
The effect of clozapine treatment on production of inflammatory cytokines in 3T3-L1, C2C12 and RAW 264.7 cells. Cells were treated with 0, 25, 50 and 75 µM clozapine for 24 hours. Cytokine levels were measured using the MILLIPLEX MAP Mouse Cytokine/Chemokine-Premixed 13 Plex array. Data shown are baseline corrected to show the relative changes in cytokine levels (in pg of cytokine per mg of protein lysate) with increasing clozapine compared to the untreated control cells. Only cytokines that are significantly altered by clozapine treatment in each cell line are shown. P values shown are based on a Bonferroni post-hoc test.

To determine whether clozapine induced the production of proinflammatory cytokines, the effect of clozapine treatment on secreted cytokine levels was determined using a multiplex array. All four cell lines showed changes in cytokine production upon clozapine treatment. Cell lines with significant alterations in cytokines levels are shown in Figure 6. In 3T3 cells, clozapine treatment led to detection of 5 analytes (IL-2, MCP-1, IL-12p70, GM-CSF, and IL-6), therefore alterations were considered significant when ANOVA analyses showed p<0.01. IL-2 (p = 0.004) and IL-6 (p = 0.009) levels were significantly altered with treatment. Levels of IL-2 increased, relative to controls, 142% (p<0.05) and over 1000% (p<0.01) at 50 and 75 µM clozapine, respectively. Levels of IL-6 decreased 90% at 25 µM clozapine relative to controls (p<0.05) and returned to control levels at 75 µM clozapine, increasing 640% (p<0.05) relative to the 25 µM dose. In C2C12 cell culture supernatants, GM-CSF, IL-6, MCP-1 and IL-12p70 were detected after treatment with clozapine. Therefore, cytokine alterations in C2C12 cells were considered significant when ANOVA analyses showed p<0.0125. GM-CSF (p = 0.005), IL-6 (p = 0.01), and IL-12p70 (p = 0.002) were significantly altered with clozapine treatment. GM-CSF was elevated 216% at 25 and 350% at 75 µM clozapine (p<0.05 and p<0.01, respectively) relative to the controls. IL-6 was elevated 880% (p<0.05) at 75 µM clozapine relative to the control, and 470% relative to the 25 µM dose (p<0.05). IL-12p70 was elevated 1000% (p<0.05) and 538% (p<0.05) at the 50 µM and 75 µM doses, respectively, relative to the control, and 1200% (p<0.05) and 545% (p<0.05) relative to the 25 µM dose. In RAW cells, several cytokines were detected in culture supernatants following clozapine treatment, including GM-CSF, IL-1b, IL-12p70, IL-10, IL-13, TNF-α, and MCP-1. Cytokine alterations in RAW supernatants were considered significant when ANOVA analyses showed p<0.007. GM-CSF (p = 0.001), IL-1b (p<0.0001), IL-12p70 (p = 0.004), and IL-13 (p<0.0001) were significantly altered with clozapine treatment. At 75 µM clozapine, GM-CSF levels were 2550% (p<0.01) higher relative to the control, which was 160% (p<0.01) and 676% (p<0.05) higher than the 25 and 50 µM doses, respectively. IL-1b levels were 5100% (p<0.0001) higher at 75 µM clozapine relative to the control. Relative to the 25 and 50 µM doses, at 75 µM clozapine, RAW cells produced 3250% (p<0.0001) and 1774% (p<0.001) more IL-1b, respectively. At 75 µM clozapine, IL-12p70 levels were 484% (p<0.05) higher relative to the control, which was 750% (p<0.01) and 463% (p<0.05), respectively, higher than the 25 and 50 µM doses. IL-13 increased 840% (p<0.001) at 75 µM clozapine relative to the control. This increase was 1771% (p<0.001) and 738% (p<0.001) higher than the 25 and 50 µM doses, respectively. MCP-1 levels were also significantly altered with clozapine treatment (p = 0.0005). MCP-1 levels decreased 76% (p<0.001) and 46% (p<0.05) at 50 µM clozapine relative to the control and the 25 µM dose, respectively. In the FL83B cell supernatants, there were detectable levels of GM-CSF, IL-6, and MCP-1 following clozapine treatement. The changes in cytokine levels were not significant, since the ANOVA analyses did not achieve p<0.017.

## Discussion

Previous studies of how atypical antipsychotics such as clozapine may give rise to increased risk for MetS and diabetes have focused primarily on the brain or brain cells, and on understanding this phenomenon with regard to psychiatric patients. In this study, in addition to SKNSH neuroblastoma cells, we examined the effect of clozapine on insulin responsive cell types and cells associated with obesity: fat, muscle, liver and inflammatory cells. These cell lines provide a simple model system for understanding the mechanistic details of clozapine-induced metabolic changes. Specifically, this study examined the effect of clozapine on mitochondrial functions and inflammation in these cell types as both mitochondrial dysfunction and inflammation are thought to give rise to obesity, insulin resistance and other symptoms of MetS [26], [27], [28]. Clozapine induced alterations in mitochondria morphology in all cell types tested (Figures 1 and 2). These alterations included changes from a contiguous mitochondrial framework to smaller punctuate pattern, and increases in overall mitochondria volume. Importantly, alterations in mitochondrial size and density have been observed in the skeletal muscle of both genetically-induced, or diet-induced obese mice [40] and in individuals with metabolic disease [33], supporting the hypothesis that clozapine-induced alterations in mitochondria morphology may be associated with the metabolic symptoms observed in patients using clozapine.

Mitochondrial turn over, morphology and remodeling varies in different cell types [41], [42], wherein mitochondria exist in two states, “individual state” and “network state” which we refer to as a punctate/fissioned or contiguous/filamentous state, respectively, in our findings [42], [43]. Importantly, within each cell, mitochondrial population can be functionally and morphologically heterogeneous based in part on the demand of oxidative phosphorylation and ATP output [41], [43] and on the internal pool of healthy and old/sick mitochondria (though there is no biomarker yet to detect the two subtypes). Therefore, any external trigger leading to cellular oxidative stress [44] can disrupt the homeostasis of proteins and transcription factors that control the process of mitochondrial fusion and fission, thus changing the mitochondrial number, distribution pattern, size and shape [43], which may lead to development of diseases such as diabetes [44].

Clozapine also caused mitochondria depolarization in all cell types examined. In most cases, there appeared to be two distinct populations of mitochondria, based on their membrane potential. Other studies have shown that reticular mitochondria have varying potential across their framework [45]. This aspect was all the more evident with increased dosing regimens of clozapine, when the reticular mitochondrial distribution is lost. Moreover, our present findings suggest that fluctuations in mitochondrial membrane potentials need stringent examination, and a single value of average membrane potential/cell may be insufficient. The depolarization of the mitochondria membrane potential is associated with an increase in mitochondria swelling [46], which we observed after clozapine treatment in all cell types investigated. The observed clozapine-induced swelling of mitochondria may be a mechanism by which cells prevent oxidative damage due to increased generation of mitochondrial ROS (mROS) by the respiratory chain. Several studies have shown that clozapine induces production of ROS [11], [47]. Other studies have shown that minor oxidative stress induces mitochondrial swelling and the formation of a mitochondrial “firewall” which prevents propagation of mROS [48]. In addition, a known cell survival strategy involves mild uncoupling of mitochondria leading to weak mitochondrial depolarization, without causing cell death [49], [50]. This may be true here as the cell adapts to clozapine exposure. This protection mechanism may be the reason why no significant decreases in cell viability were observed for most of the cell lines after clozapine treatment. The observed mitochondria membrane depolarization is consistent with the observed depletion of ATP at increased concentrations of clozapine. In the monocyte cell line, this depletion of ATP was evident at the lowest dose of clozapine tested, and at the intermediate concentration of clozapine in the preadipocyte and liver cell lines, suggesting that lower levels of clozapine are capable of severely disrupting energy metabolism in the cell lines tested.

Alterations in mitochondria function are associated with increased inflammation. Increased production of proinflammatory cytokines was observed after clozapine treatment. Inflammation has been shown to play a key role in obesity and diabetes [39]. In fact, the pathogenesis of diabetes and its metabolic complications exist in a state of chronic systemic inflammation. Such chronic inflammation increases the expression of circulating inflammatory factors. Atypical antipsychotics have been reported to be both protective against inflammation, as well as causative [51]. However, these studies have been performed on different cell types, under different conditions, and have not been systematically conducted. While there is some debate about the inflammatory nature of clozapine, there is evidence to support that this drug influences the production of several cytokines and/or cytokine receptors that modulate immunological responses [20], [21], [22]. In support of this, we observed here that clozapine induces elevated production of proinflammatory cytokines/chemokines in adipocyte, muscle, and monocyte cell lines. Of particular interest is the robust proinflammatory response in monocytes, which includes the production of IL-1b, a cytokine whose production is known to be triggered by the inflammasome [23], [24], a multi-protein complex which initiates an inflammatory response in response to mitochondria dysfunction or reactive oxygen species. The findings of a clozapine-induced “pro-inflammatory” state in monocytes is important, as monocyte infiltration of adipose and other tissue, followed by the local production of proinflammatory cytokines, has been found to be associated with obesity and insulin resistance [52]. One such cytokine is IL-6, whose production was altered in response to clozapine in adipocyte and muscle cells. Circulating IL-6 elicits many types of responses, and has been found to correlate with insulin resistance and altered carbon metabolism [53]. Recently it was shown that decreasing inflammation and monocyte infiltration to muscle and liver tissue could reduce the development of MetS in a mouse model [54]. The findings herein suggest that a variety of cell types are susceptible to a clozapine-induced proinflammatory state that may promote cellular dysfunction. In patients, this may be further exacerbated by monocyte-infiltration of tissues resulting in further local inflammation.

In summary, the findings of clozapine-induced mitochondrial and inflammatory alterations in insulin responsive cells support the aforementioned link between mitochondria function and inflammation in risk for MetS, and suggest that alterations in these pathways may underlie the causes of clozapine-induced MetS. It is important to note that, as this was an exploratory study to determine whether a DIMS/cell culture model system could produce cellular and molecular phenomena biologically relevant to MetS, control drugs were not included in the design. Our findings show that a cell-culture-DIMS-based approach might be a useful tool for achieving a better mechanistic understanding of the genesis of diabetes and MetS, and that such a tool is but one of many that might be used to fully understand metabolic disease. Further studies with other antipsychotics, both typical and atypical, including those which are not known to cause increased risk for weight gain or MetS, should be performed in order to determine if the current observed effects are unique to clozapine or to atypical antipsychotics and could therefore indeed 1) be causative of the unique clinical side-effects seen with these drugs and 2) identify relevant cellular and molecular mechanisms which may give rise to MetS.

## Materials and Methods

### Cell Culture and Clozapine Treatment

All cell lines were obtained from the ATCC (Manasas, VA, USA). SKNSH human neuroblastoma cells were cultured in DMEM supplemented with 4 mM L- glutamine as previously described [11]. The mouse cell lines, 3T3-L1 (3T3) preadipocytes, C2C12 myoblasts, and RAW 264.7 (RAW) monocytes, were cultured in DMEM supplemented with 110 mg/L sodium pyruvate, 4 mM L-glutamine and 10% fetal bovine serum. FL83B mouse hepatocytes were cultured in F-12 K media containing 10% fetal bovine serum. To determine the effects of clozapine treatment on SKNSH cells, cells were treated with 10, 20 and 50 µM clozapine or vehicle (0.65% DMSO only) for 24 hours. To determine the effects of clozapine treatment on the other cell lines, cells were treated with 25, 50 and 75 µM clozapine or vehicle (0.65% DMSO only) for 24 hours. All assays described below were performed in triplicate at each concentration.

### Cell Viability

The effect of clozapine on the viability of RAW, C2C12, 3T3 and FL83B cells was determined by neutral red assay (Sigma, St. Louis, MO, USA). Only viable cells are capable of incorporating neutral red dye by active transport. Briefly, after 24 hours of clozapine treatment or vehicle, the cells were rinsed with PBS and incubated in media containing 0.033% neutral red for two hours. Cells were then washed several times with PBS and the incorporated neutral red dye was solubilized by gentle rocking for 10 minutes with a solution of 1% acetic acid and 50% ethanol. After 10 min, the solution was collected and the amount of incorporated neutral red dye was determined spectrophotometrically by measuring the absorbance of the solution at 540 nm.

### Mitochondria Morphology and Membrane Potential

To visualize mitochondria morphology and measure the effects of clozapine on membrane potential after 24 hours of treatment, cells were cultured on nunc chambers and incubated with media containing 30 nM tetramethylrhodamine methyl ester perchlorate (TMRM; Invitrogen, Eugene, OR, USA) for 30 min. Z-series confocal images of the cells were then obtained using a FV1000 imaging system mounted on an Olympus IX-81 inverted microscope using a Plan-Achromat 63x/1.4 Oil DIC objective. To reduce phototoxicity, laser intensity was kept at the lowest for excitation of TMRM but sufficient for cell imaging. Laser power settings for each cell type were maintained constant throughout the experiment. The collected Z-image series were used to examine the appearance of mitochondria for the characteristic reticular morphology of normal mitochondria, the presence of numerous round fragments of varying size indicative of fission, or filamentous elongation indicative of fusion. The images were further analyzed to measure mitochondrial membrane potential and volume using the Nernst Potential MulPro2D plug-in for Image J software (National Institute of Health, USA). The plug-in identifies individual mitochondria and, using the fluorescence of TMRM and the Nernst Equation, calculates the mitochondrial membrane potential for each identified mitochondria.

### ATP Levels

To determine the effect of clozapine treatment on ATP production, ATP levels were determined by bioluminescence assay (Roche Applied Science, Indianapolis, IN, USA) which measures the ATP-dependent conversion of luciferin to oxyluciferin and light. After 24 hours of clozapine or vehicle only treatment, cells were washed with PBS and an ATP lysate was made for each replicate culture using boiling lysis buffer or boiling water. ATP levels for each replicate were measured in duplicate. Lysates were combined with luciferase reagent per the manufacturer's instruction, and the resulting light emission at 562 nm was quantified by a microplate-format luminometer. ATP lysates were also quantified for A260 by NanoDrop (Thermo Scientific, Wilmington, DE, USA). The luminescence values were further corrected by using A260 values to correct for the number of viable cells contributing to ATP levels.

### Cytokine Analysis by Luminex Assay

To determine the effect of clozapine treatment on the production of proinflammatory cytokines, cell culture supernatants from treated and control cells were analyzed for 13 different cytokines using a using a multiplex Luminex bead-based assay (MILLIPLEX MAP Mouse Cytokine/Chemokine-Premixed 13 Plex; Millipore, Billerica, MA, USA) capable of detecting the following analytes: GM-CSF, IFN-γ, IL-10, IL-12 (p70), IL-13, IL-1β, IL-2, IL-4, IL-5, IL-6, IL-7, MCP-1, and TNF-α. After 24 hours of clozapine treatment, culture media from clozapine-treated and control cells was collected, aliquoted and stored at −80°C until assayed according to the manufacturer's instructions. Briefly, prior to the assay, culture supernatants were concentrated 2- to 3-fold using microconcentrators (Corning Spin-X UF Concentrators; Sigma, St. Louis, MO, USA) with a 10 K molecular weight cut-off. Protein content of culture supernatants was quantified for A280 by NanoDrop before and after concentrating. Cytokine levels for each drug dose replicate were measured in duplicate. Equivalent amounts of concentrated culture supernatant samples were incubated with antibody-coated capture beads overnight at 4 °C. Washed beads were further incubated with biotin-labeled anti-mouse cytokine antibodies for 1 h at room temperature followed by incubation with streptavidin-phycoerythrin for 30 min. Samples were analyzed using Luminex 200™ (Luminex, Austin, TX, USA) and Statlia software (Brendan Technologies Inc., Carlsbad, CA, USA). Standard curves of known concentrations of recombinant mouse cytokines were used to convert median fluorescence intensity (MFI) to cytokine concentration in pg/ml and these were further corrected by cell lysate protein concentration to correct for the number of viable cells contributing to cytokine levels. For this, cell lysates were prepared from the monolayer from which the culture supernatants were collected. These cells were washed with PBS and lysed using ice cold RIPA buffer. Lysates were quantified by BCA Assay (Thermo Fisher Scientific, Rockford, IL, US*A*).

### Statistical Analyses

Logarithmic (base 10) transformations were applied to the absolute mitochondrial volume data values obtained from NIH Image J analysis. The data was evaluated for a normal distribution using the Shapiro-Wilks test and the Kolmogorov-Smirnov test. Plots for comparing Nernst mitochondrial membrane potential distribution across treatments were drawn by adding Gaussian kernel density plots to the histograms. Comparisons of mitochondrial Nernst potential and mitochondrial volume values were made to their respective controls, and performed by non-parametric Rank-sum and Kruskal Wallis Tests, where for multipurpose comparisons a p<0.016 is considered significant. Statistical analysis and graphics were made using Stata 11.0 (Stata Corp, College Station, TX). Other statistical analyses were performed using the software SPSS (IBM, Armonk, NY, USA) or GraphPad Prism (GraphPad Softwars, La Jolla, CA, USA). Data is expressed as the mean ± SEM. Differences in the end-points (variables) between doses of clozapine were determined by an analysis of variance (ANOVA) incorporating repeated measures across dose. For the analyses of cytokine levels, data was log transformed and ANOVA was performed using additional post-hoc analyses to define significant relationships between the individual end-points. To correct for multiple comparisons, for each cell line, statistical significance was determined using Bonferroni correction, where p = 0.05 was divided by the number of hypothesis tested (i.e., the number of cytokines detected in each cell line). Therefore, as discussed in the results, the p value considered to be significant was different for each cell line.

## Supporting Information

Table S1 **Change in membrane potential after treatment with clozapine.**
(DOCX)

Table S2 **Mitochondria membrane potential and volume after 24 hr clozapine treatment.**
(DOCX)
