# Supplementary material for: Clozapine-Induced Mitochondria Alterations and Inflammation in Brain and Insulin-Responsive Cells
Source: PLoS One. 2013 Mar 20;8(3):e59012. doi: 10.1371/journal.pone.0059012 (PMC3604003; doi:10.1371/journal.pone.0059012)
Supplement: Table S1 — Change in membrane potential after treatment with clozapine. (DOCX) [file pone.0059012.s001.docx]

**Table S1. Change in membrane potential after treatment with clozapine.**

| **SHSY** |  |  |  |  |  |  |
| --- | --- | --- | --- | --- | --- | --- |
| **Threshold** | **N** | **% above -120** | **% above -130** | **% above -140** | **% above -150** | **% above**  **Control Median**  **(-147.43)** |
| **Control** | 332 | 1.80 | 5.12 | 24.39 | 59.63 |  |
| **Clozapine 10 μM** | 353 | 1.69 | 10.76 | 21.52 | 53.82 | 47.02 |
| **Clozapine 20 μM** | 275 | 19.63 | 49.45 | 65.09 | 88.72 | 84.72 |
| **Clozapine 50 μM** | 416 | 8.22 | 22.35 | 48.07 | 75.48 | 72.11 |
| **Chi2** |  | 93.8175 | 209.9797 | 168.2126 | 109.2877 | 133.0238 |
| **P value** |  | <0.001 | <0.001 | <0.001 | <0.001 | <0.001 |
| **3T3** |  |  |  |  |  |  |
| **Threshold** | **N** | **% above**  **-120** | **% above**  **-130** | **% above -140** | **% above**  **-150** | **% above**  **Control Median**  **(-148.08)** |
| **Control** | 952 | 0.1 | 3.57 | 22.58 | 57.66 |  |
| **Clozapine 25 μM** | 912 | 4.38 | 4.38 | 4.60 | 10.30 | 6.90 |
| **Clozapine 50 μM** | 462 | 11.47 | 32.46 | 32.90 | 46.96 | 42.20 |
| **Clozapine 75 μM** | 340 | 43.52 | 57.94 | 62.64 | 82.35 | 81.47 |
| **Chi2** |  | 609.3569 | 746.9842 | 496.5407 | 699.9813 | 709.1371 |
| **P value** |  | <0.001 | <0.001 | <0.001 | <0.001 | <0.001 |
| **C2C12** |  |  |  |  |  |  |
| **Threshold** | **N** | **% above**  **-120** | **% above**  **-130** | **% above -140** | **% above**  **-150** | **% above**  **Control Median**  **(-131.06)** |
| **Clozapine Control** | 1001 | 31.86 | 47.24 | 71.22 | 91.80 |  |
| **Clozapine 25 μM** | 815 | 2.33 | 26.87 | 63.92 | 87.73 | 31.90 |
| **Clozapine 50 μM** | 1344 | 39.65 | 64.65 | 84.74 | 94.27 | 67.63 |
| **Clozapine 75 μM** | 371 | 45.82 | 82.47 | 97.30 | 100 | 84.09 |
| **Chi2** |  | 406.3046 | 439.2501 | 232.0839 | 64.7483 | 399.8293 |
| **P value** |  | <0.001 | <0.001 | <0.001 | <0.001 | <0.001 |
| **FL83B** |  |  |  |  |  |  |
| **Threshold** | **N** | **% above**  **-120** | **% above**  **-130** | **% above -140** | **% above**  **-150** | **% above**  **Control Median**  **(-143.16)** |
| **Clozapine Control** | 272 | 4.77 | 17.27 | 43.75 | 61.39 |  |
| **Clozapine 25 μM** | 375 | 0 | 2.4 | 19.73 | 33.06 | 24.8 |
| **Clozapine 50 μM** | 603 | 2.48 | 21.72 | 45.93 | 65.00 | 52.40 |
| **Clozapine 75 μM** | 860 | 14.30 | 49.91 | 68.02 | 91.27 | 76.51 |
| **Chi2** |  | 117.0960 | 346.0912 | 680.4292 | 442.0212 | 302.8730 |
| **P value** |  | <0.001 | <0.001 | <0.001 | <0.001 | <0.001 |
| **RAW** |  |  |  |  |  |  |
| **Threshold** | **N** | **% above**  **-120** | **% above**  **-130** | **% above -140** | **% above**  **-150** | **% above**  **Control Median**  **(-125.71)** |
| **Clozapine Control** | 1260 | 25.23 | 67.06 | 90.55 | 99.68 |  |
| **Clozapine 25 μM** | 1770 | 18.02 | 51.01 | 78.92 | 96.97 | 37.00 |
| **Clozapine 50 μM** | 724 | 14.64 | 58.14 | 84.66 | 94.47 | 36.32 |
| **Clozapine 75 μM** | 201 | 24.87 | 54.22 | 76.61 | 95.52 | 41.79 |
| **Chi2** |  | 41.8293 | 78.6362 | 80.2693 | 51.6090 | 59.5173 |
| **P value** |  | <0.001 | <0.001 | <0.001 | <0.001 | <0.001 |

Table shows percent change in Nernst potential above indicated thresholds, and the percent distribution above the median of the control, to represent the shift in the medians after clozapine treatment. N=number of mitochondria analyzed in each case.
