# Supplementary material for: Clozapine-Induced Mitochondria Alterations and Inflammation in Brain and Insulin-Responsive Cells
Source: PLoS One. 2013 Mar 20;8(3):e59012. doi: 10.1371/journal.pone.0059012 (PMC3604003; doi:10.1371/journal.pone.0059012)
Supplement: Table S2 — Mitochondria membrane potential and volume after 24 hr clozapine treatment. (DOCX) [file pone.0059012.s002.docx]

**Table S2. Mitochondria membrane potential and volume after 24 hr clozapine treatment.**

|  | | **Nernst Potential** | | | | **Volume (log_10_)** | | | |
| --- | --- | --- | --- | --- | --- | --- | --- | --- | --- |
| **Cell line** | **N** | **Mean** | **Median** | **SD** | **IQR** | **Mean** | **Median** | **SD** | **IQR** |
| **SKNSH** |  |  |  |  |  |  |  |  |  |
| Control | 332 | -147.25 | -147.43 | 11.21 | -153.26 – -140.67 | 1.86 | 1.68 | 0.86 | 1.17 – 2.37 |
| 10 μM | 353 | -148.80 | -148.54 | 13.35 | -158.22 – -140.89 | 2.06 | 2.12 | 0.91 | 1.23 – 2.64 |
| 20 μM | 275 | -130.85 | -130.85 | 12.33 | -145.27 – -122.15 | 2.01 | 1.86 | 0.96 | 1.17 – 2.86 |
| 50 μM | 416 | -140.10 | -141.05 | 12.95 | -149.72 – -131.06 | 2.12 | 2.15 | 0.66 | 1.66 – 2.56 |
| **RAW** |  |  |  |  |  |  |  |  |  |
| Control | 1260 | -126.42 | -125.71 | 9.08 | -132.36 – -119.90 | 1.26 | 1.11 | 0.54 | 0.84 – 1.51 |
| 25 μM | 1770 | -130.72 | -129.74 | 10.81 | -138.48 – -122.25 | 1.20 | 1.07 | 0.49 | 0.84 – 1.43 |
| 50 μM | 724 | -129.84 | -128.33 | 10.02 | -135.48 – -122.86 | 1.28 | 1.11 | 0.62 | 0.84 – 1.50 |
| 75 μM | 201 | -130.29 | -128.54 | 11.86 | -138.90 – -120.07 | 1.36 | 1.23 | 0.67 | 0.95 – 1.57 |
| **3T3** |  |  |  |  |  |  |  |  |  |
| Control | 952 | -146.93 | -148.08 | 8.39 | -154.42 – -141.56 | 1.35 | 1.23 | 0.57 | 0.90 – 1.66 |
| 25 μM | 912 | -157.13 | -159.11 | 13.30 | -164.99 – -153.94 | 1.36 | 1.23 | 0.60 | 0.90 – 1.64 |
| 50 μM | 462 | -143.13 | -150.86 | 18.03 | -158.87 – -123.42 | 1.58 | 1.44 | 0.72 | 1.11 – 1.91 |
| 75 μM | 340 | -127.99 | -122.03 | 15.97 | -142.49 – -115.50 | 1.87 | 1.85 | 0.79 | 1.39 – 2.26 |
| **C2C12** |  |  |  |  |  |  |  |  |  |
| Control | 1001 | -128.29 | -131.06 | 17.10 | -141.35 – -113.54 | 1.29 | 1.14 | 0.58 | 0.90 – 1.51 |
| 25 μM | 815 | -137.27 | -135.83 | 10.50 | -144.01 – -129.26 | 1.34 | 1.17 | 0.59 | 0.90 – 1.59 |
| 50 μM | 1344 | -125.80 | -123.47 | 13.53 | -134.73 – -115.53 | 1.48 | 1.35 | 0.61 | 0.77 – 1.88 |
| 75 μM | 371 | -120.10 | -121.07 | 11.34 | -128.78 – -111.82 | 1.37 | 1.23 | 0.32 | 0.95 – 1.67 |
| **FL83B** |  |  |  |  |  |  |  |  |  |
| Control | 272 | -144.43 | -143.16 | 15.37 | -155.62 – -133.27 | 1.27 | 1.07 | 0.62 | 0.84 -1.47 |
| 25 μM | 375 | -170.52 | -170.87 | 27.24 | -195.62 – -143.37 | 1.46 | 1.30 | 0.68 | 0.90 – 1.88 |
| 50 μM | 603 | -143.73 | -142.16 | 15.23 | -155.97 – -131.15 | 1.45 | 1.27 | 0.68 | 0.84 – 1.88 |
| 75 μM | 860 | -133.86 | -134.41 | 12.38 | -142.68 – -125.70 | 1.41 | 1.30 | 0.60 | 0.95 – 1.72 |

Measurements of central tendency are represented by the mean and median. IQR and SD represent

measurements of dispersion. N= number of mitochondria analyzed in each case.
